# Co-Designing Mobile Serious Games to Support Patients With Psoriatic Arthritis and Chronic Pain: Mixed Methods Study

**DOI:** 10.2196/75072

**Published:** 2026-01-30

**Authors:** Bárbara Ramalho, Samuel Gomes, Marta Silva Vicente, Filipa Magalhães, Rodolfo Gonçalves Costa, Sandra Gama, Vasileios Charisis, Leontios Hadjileontiadis, Sofia B Dias, iPROLEPSIS Consortium

**Affiliations:** 1 HUMAN Lab, INESC-ID, Instituto Superior Técnico, Faculdade de Motricidade Humana, Universidade de Lisboa, Lisbon, Oeiras, Portugal; 2 HUMAN Lab, INESC-ID, Instituto Superior Técnico, Universidade de Lisboa, Lisbon, Portugal; 3 Department of Electrical and Computer Engineering, Aristotle University of Thessaloniki, Thessaloniki, Central Macedonia, Greece; 4 Department of Biomedical Engineering and Biotechnology, Khalifa University, Abu Dhabi, United Arab Emirates; 5 Center of Interdisciplinary Study of Human Perfomance (CIPER), Faculdade de Motricidade Humana, Universidade de Lisboa, Estrada da Costa, 1495-751 Cruz Quebrada, Lisbon, Portugal, 351 939576909

**Keywords:** serious games, psoriatic arthritis, chronic pain, NoPain Games, mHealth, digital health care, iPROLEPSIS, mobile health

## Abstract

**Background:**

Serious games offer promising avenues for clinical care by enhancing patient engagement and delivering therapeutic benefits. In psoriatic arthritis (PsA), chronic pain contributes to emotional distress, functional limitations, and reduced well-being. While symptom-tracking apps exist, few digital interventions directly address chronic pain through engaging, therapeutic experiences tailored to patients’ cognitive and physical needs.

**Objective:**

This study aimed to co-design mobile serious games*—*NoPain Games—to support patients with PsA in managing chronic pain. Conducted within the iPROLEPSIS Horizon Europe project, the study involved a multidisciplinary cocreation session with rheumatologists, researchers, and technical experts, followed by a usability feedback session with patients with PsA. The goal was to identify therapeutic priorities, refine game mechanics, and assess usability to inform the development of personalized, accessible digital interventions.

**Methods:**

A sequential mixed methods design was used. First, a 90-minute remote cocreation session was held with 14 experts (6 rheumatologists, 4 technical experts, and 4 researchers) from 3 European countries. Participants reviewed game storyboards and discussed therapeutic and design priorities. Thematic analysis of transcribed discussions identified key insights. Next, a 60-minute remote usability feedback session was conducted with 5 patients with PsA (aged 25‐64 y), who interacted with 2 high-fidelity game prototypes (Space Oddity and Four Seasons). Usability was assessed using the System Usability Scale (SUS), and qualitative feedback was collected through moderated discussion. Item-level analysis using item characteristic curves provided deeper insight into usability perceptions and item sensitivity. All ethics requirements were met for both study phases.

**Results:**

Thematic analysis of the transcribed dialogs of the cocreation session revealed three core themes: (1) therapeutic benefits (pain distraction, memory enhancement, cognitive stimulation, stress reduction, and creative engagement); (2) game difficulty (balancing duration and complexity to sustain engagement without fatigue); and (3) accessibility and interaction (addressing physical limitations, optimizing touchscreen usability, and ensuring inclusive design). These insights informed the development of 2 NoPain Game prototypes, which received a SUS score of 79 (SD 10.4; 95% CI 69.89‐88.11), indicating good usability. Item characteristic curve analysis showed strong discrimination for learnability, while ease of use and confidence exhibited ceiling effects. Items like support needs and inconsistency showed minimal variability, and the learning curve demonstrated delayed but meaningful responsiveness at higher usability levels. Qualitative feedback reinforced the relevance of difficulty adjustment, technical refinements, and game mechanics, offering actionable insights for future iterations and broader implementation.

**Conclusions:**

This study uniquely contributes to the field by co-designing mobile serious games specifically for patients with PsA, integrating expert and patient input to address chronic pain through accessible, cognitively engaging digital interventions—an approach not previously explored in this population. Future work will refine game mechanics, integrate adaptive difficulty, and conduct clinical trials to evaluate therapeutic efficacy. NoPain Games may serve as complementary tools within digital care ecosystems, offering support tailored to the therapeutic needs and physical limitations of patients with PsA underserved by conventional therapies.

## Introduction

In health care, serious games—designed for purposes beyond entertainment—have generated considerable interest and sparked ongoing debate [1]. Their potential has often been overlooked due to the still prevailing perception of games as purely recreational objects rather than valuable tools for skill development and health rehabilitation [2]. Research studies, however, have demonstrated the benefits of serious games, particularly their ability to reduce stress [3], enhance mobility [4,5], and alleviate pain [6,7].

Many of these benefits align with the symptomatology of rheumatic diseases, making it possible to use serious games as health care tools for conditions such as psoriatic arthritis (PsA). PsA, a degenerative rheumatic disease, is marked by joint stiffness and persistent pain, significantly affecting patients’ quality of life [8]. As there is no cure, treatment primarily focuses on managing symptoms and holistically improving patients’ well-being, often through interventions like physical therapy [9]. However, these traditional approaches can feel repetitive or burdensome for patients, leading to low adherence and reduced effectiveness. While various digital tools, particularly mobile apps for disease management, have been proposed [10], they primarily serve as symptom-tracking solutions and fail to address the ongoing joint pain experienced by patients.

Serious games offer a compelling alternative to complement traditional treatments by creating experiences that are both engaging and therapeutic [11]. For example, DaktylAct, a touch-based serious game, has been proposed as an innovative tool for assessing fine motor skills in PsA using novel digital biomarkers [12]. Furthermore, immersive experiences in chronic pain management have been extensively studied, with strong evidence supporting their pain-relieving capabilities, particularly in virtual reality (VR) environments [6,13]. Building on this, previous research has highlighted the effectiveness of exergames in altering pain perception by engaging multiple sensory modalities [14]. For instance, Gold et al [13] highlighted how VR can influence neurobiological mechanisms, showing that auditory, visual, and tactile stimuli reduce activity in pain-processing areas. In addition, Hoffman et al [15] further validated the effectiveness of immersive digital interventions by comparing VR and opioids in pain relief. Their findings suggest that while both approaches are beneficial independently, their combination leads to even greater analgesic effects. These findings, derived from subjective pain scales and functional magnetic resonance imaging, highlight the potential of mobile-based alternatives with similar engagement mechanisms to perform effectively without the need for specialized hardware.

Moreover, recent studies have further expanded the evidence base for serious games in pain management. Beltran-Alacreu et al [16] developed a task-oriented serious game for older adults with chronic neck pain, demonstrating its suitability and therapeutic potential. Saragih et al [7] conducted a systematic review and meta-analysis confirming the efficacy of serious games in managing chronic pain among older adults, reinforcing their clinical relevance. Additionally, Peña et al [17] explored the use of digital art and attachment priming in a web-based serious game, showing promising results in reducing both pain and social disconnection in individuals with chronic pain and loneliness.

Immersion remains a critical factor in pain management games. Gromala et al [6] emphasized that realistic visual environments, sound effects, and interactive storytelling contribute significantly to the overall therapeutic experience. Their concept of imaginary immersion introduces in-game threats and challenges to sustain player focus and engagement. We believe that translating these principles into mobile platforms requires innovative game mechanics that maintain immersion despite limited sensory channels. For instance, the MyRelief smartphone app presents a serious game to relieve chronic low-back pain through physical and psychological activities [18]. Although their analysis suggests that serious games can help improve patient status, the authors recognize that further research is needed to validate all game components.

Incorporating motivational elements in mobile pain management games is another critical design consideration. In this line, Ijaz et al [19] demonstrated the power of competitive motivation in their cycling game, which used on-screen scores of real and artificial competitors to enhance user engagement. Similarly, Tuah et al [20] reviewed common gamification elements in rehabilitation games, identifying key components such as points, leaderboards, badges, progression systems, and avatars that contribute to sustained participation.

To the best of our knowledge, no serious game has been specifically designed to target the chronic pain experienced by individuals with PsA. This study aims to explore the co-design and development of mobile serious games, referred to as NoPain Games, specifically tailored to support patients with PsA in managing chronic pain. Developed using an agile co-design methodology [21], these serious games represent a new approach to chronic pain management, as part of the Horizon Europe iPROLEPSIS project [22,23], which aims to create a personalized suite of games tailored to the needs of individuals with PsA. The primary objective of this study is to assess the therapeutic potential, usability, and design requirements of these games through a multidisciplinary cocreation process involving clinicians, researchers, and technical experts, along with the patients’ usability feedback. By integrating cognitive stimulation, stress reduction, and inclusive interaction mechanics, the study seeks to establish foundational design principles for mobile-based digital interventions that enhance patient engagement and well-being. The underlying hypothesis is that co-designed serious games incorporating adaptive difficulty and accessible interfaces can serve as effective pain distractors and cognitive enhancers for individuals living with PsA.

## Methods

### Overview

In this study, we followed a sequential mixed methods design, integrating qualitative and quantitative data to inform the co-design and evaluation of mobile serious games for patients with PsA. This sequential design was selected to allow expert-derived qualitative insights to inform prototype development prior to patient usability testing. Integration occurred through iterative refinement of game mechanics based on themes from the cocreation session, followed by triangulation of usability scores and patient feedback to validate design decisions. In the qualitative phase, 14 experts (6 rheumatologists, 4 technical experts, 4 researchers) were recruited via purposive sampling from the iPROLEPSIS Consortium and participated in a 90-minute remote cocreation session. Eligibility required domain expertise in rheumatology, digital health, or game development. Participants reviewed game storyboards and contributed to design refinement through moderated discussion. In the quantitative phase, 5 patients with PsA (age 25‐64 years) from 4 countries were recruited through targeted outreach. Eligibility required a confirmed PsA diagnosis and the ability to interact with mobile devices. Patients participated in a 60-minute remote usability session, interacting with 2 high-fidelity game prototypes. Usability was assessed using the System Usability Scale (SUS), and qualitative feedback was collected through a structured discussion. Study outcomes included: (1) identification of therapeutic priorities and design requirements (qualitative), (2) usability scores and item-level sensitivity analysis (quantitative), and (3) patient feedback on game experience and accessibility (qualitative). Thematic analysis followed Braun and Clarke’s framework, and SUS data were analyzed using descriptive statistics and item characteristic curves (ICCs). The study received ethical approval, and informed consent was secured from all participants.

The following subsections provide a detailed account of the study context, co-design procedures, participant characteristics, data collection instruments, ethics, and analytic strategies used to generate and interpret the findings.

### Study Context

This study is part of the iPROLEPSIS Horizon Europe research project, which seeks to investigate the progression from general health to PsA through multi-source data analysis, ultimately creating an innovative, personalized digital care ecosystem. Within the framework of iPROLEPSIS, the project focuses on designing, developing, and validating cutting-edge digital biomarkers to assess and address PsA using an Artificial Intelligence-Personalized Game Suite (AI-PGS). Developed collaboratively with key stakeholders, the AI-PGS takes a comprehensive, multitargeted approach to managing PsA symptoms. It includes intervention activities aimed at enhancing breathing, mobility, stiffness, balance, coordination, fitness, diet, and mood. By embracing a holistic perspective, the AI-PGS addresses stress, anxiety, fatigue, and pain through various categories of serious games, such as NoPain Games, Exercise Games [24], Sensorimotor Art Games [25], Breathing Games [26], Dietary Games, and Emotional Games. This study emphasizes NoPain Games, showcasing the critical role of engaging patients in therapeutic activities that incorporate cognitive processes—such as memory, coordination, and visual perception tasks—to help alleviate and manage chronic pain and discomfort associated with PsA symptoms.

### NoPain Games Agile Co-Design Process

**Figure 1.**
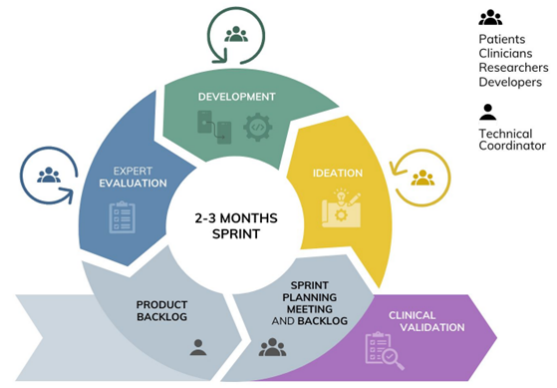
Schematic representation of the development framework for the proposed NoPain Games, illustrating the cocreation and agile methodologies used to achieve a minimum viable product. The process includes iterative 2‐3 month sprints involving product backlog refinement (led by a technical coordinator), sprint planning, ideation, game development, expert evaluation, and clinical validation. All phases, except product backlog refinement, actively engaged patients, clinicians, researchers, and developers to ensure a multidisciplinary and user-centered approach.

**Figure 2.**
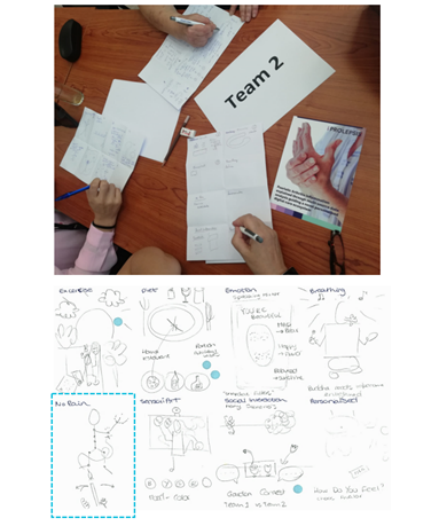
Co-design session involving patients with psoriatic arthritis during the ideation process of the games using Crazy8s method. The initial sketch of the NoPain Games category is marked with a dashed blue square.

**Figure 3.**
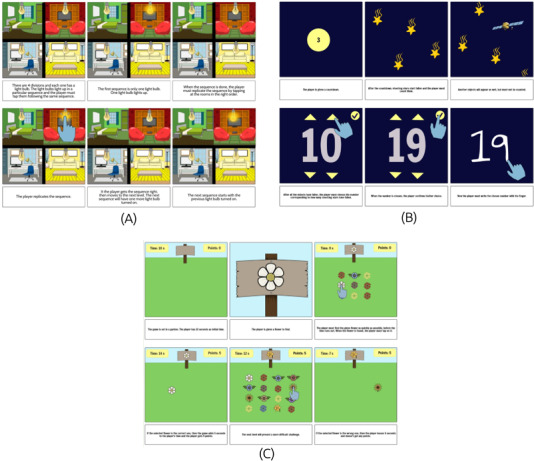
Screenshot of the storyboards for the proposed NoPain games: (A) The “Lightning” game illustrates a memory challenge where players light up rooms to replicate a specific pattern; (B) The “Flashing Stars” game presents a relaxation activity focused on counting falling stars; and (C) “The Garden” game features a calming experience in which players identify specific flowers in a vibrant field.

**Figure 4.**
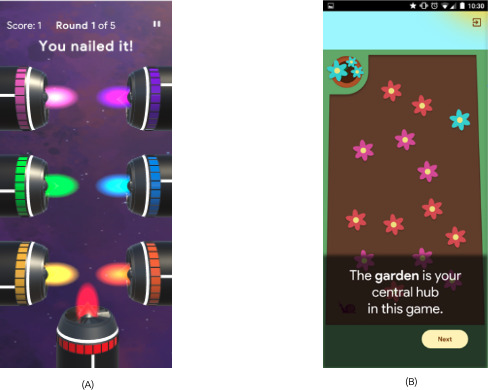
Screenshot of the prototypes used in the usability evaluation, featuring (A) the “Space Oddity” rockets minigame and (B) “The Four Seasons” design prototype.

The design and development process for the proposed games followed an agile approach [21], emphasizing early feedback to iteratively refine game concepts and prototypes. The ideation and storyboards phase will guide the foundation for subsequent stages, including prototyping, software development, and expert evaluation, while ensuring continuous cocreation efforts aimed at clinical validation (see Figure 1). Following the product backlog, sprint planning meetings, and backlog refinement phases, the design process began with the Crazy8s ideation approach [27]. More specifically, stakeholders—including patients, clinicians, researchers, and technicians—collaboratively sketched ideas for various game categories within the AI-PGS framework, including NoPain Games (see Figure 2). This phase captured diverse requirements and expectations, culminating in the initial development of a game design document outlining the NoPain Games’ key aspects, such as their concepts, mechanics, design, controls, and clinical relevance. This study highlights the subsequent agile cocreation sessions that brought together health care professionals, researchers, and technical experts as active cocreators and co-designers (see “Study participants and data collection” section) and patients with PsA as usability feedback providers (see “Feedback From Patients With PsA” section). In particular, using the storyboard technique [28], a collaborative session focused on refining and assessing the proposed designs for the NoPain Games (Figure 3), while another later session focused on the usability and general feedback of functional prototypes (Figure 4).

### Initially Proposed NoPain Games Storyboards

Three different NoPain Games were initially designed: “Lightning,” “Flashing Stars,” and “The Garden” with related storyboards (Figure 3). In the “Lightning” game, players/patients are required to replicate a given pattern by lighting up rooms in a 2D house, reinforcing memory and coordination (Figure 3A). The “Flashing Stars” game engages players in counting falling stars within a night-sky scenario, fostering relaxation through gentle visual and cognitive stimulation (Figure 3B). Finally, “The Garden” game involves identifying a specific flower type within a vast field of blooms, promoting a sense of calm and concentration (Figure 3C). Overall, by incorporating the repetitive and rhythmic interactions of identifying flowers, lighting up rooms, or counting falling stars, the proposed NoPain Games encourage users to engage in predictable, structured movements that promote relaxation and a sense of control. These are simple and rewarding mechanics aimed to provide immediate feedback, reinforcing positive engagement, and promoting a sense of accomplishment, while enabling a calm environment. Designed for both iOS and Android operating systems, the proposed NoPain Games high-fidelity prototypes present similar interaction controls, with players/patients using a touchscreen for interaction, with calming audio to match each scenario.

### Study Participants

#### Cocreation Session

To obtain feedback on the related storyboards, a cocreation session was held. The latter occurred in October 2023 and was conducted digitally via the Microsoft Teams platform with shared storyboard visuals. The session included 14 experts from 3 European countries, namely the United Kingdom, Greece, and Portugal. Participants were selected using purposive sampling [29], intentionally inviting individuals with expertise in relevant fields. They were identified through institutional affiliations within the iPROLEPSIS Consortium and invited via email by the coordinating team. Participation was based on voluntary engagement and without the provision of compensation. Exclusion criteria for experts included a lack of direct experience with PsA or digital health interventions. The group included 6 rheumatologists, 4 researchers, and 4 technical experts from the iPROLEPSIS Consortium, with multidisciplinary perspectives, enriching the discussions. The cocreation session was moderated by the last author, a qualitative researcher with expertise in participatory design. To minimize bias, the moderator did not participate in game development. Two qualitative coders (first and last author) independently reviewed transcripts and discussed potential biases during theme development (see Results).

#### Feedback Session

To maintain a patient-centered and clinically relevant design process, individuals living with PsA were actively engaged in a dedicated feedback session conducted in May 2025. During the session, participants evaluated a pilot version of Space Oddity and a design prototype of Four Seasons (Figure 4). A 60-minute remote feedback session was conducted via Zoom with 5 patients with PsA from Portugal, the United Kingdom, the Netherlands, and Greece, using screen sharing for gameplay and an embedded SUS questionnaire via Google Forms. Similar to the cocreation session, participants were recruited through targeted outreach within the iPROLEPSIS network, through clinician referrals and patient advocacy networks affiliated with the project, using email and phone outreach. Participation was based on voluntary engagement and without the provision of compensation. For patients with PsA, exclusion criteria included cognitive impairments, severe visual/motor limitations preventing touchscreen interaction, or the inability to participate in digital sessions. The feedback session was also moderated by the last author, who did not participate in the quantitative analysis to minimize bias in the findings.

### Data Collection

#### Cocreation Session

Audio recordings of the cocreation session were transcribed verbatim and then pseudoanonymized to preserve participant confidentiality. The digital setting facilitated active engagement from all attendees. The session was led by a female researcher specializing in qualitative research (last author), who guided discussions by posing questions and fostering group interactions to gather participants’ opinions and experiences. To enhance participants’ understanding of the session topics, relevant storyboards for each NoPain game were presented (Figure 3). The session followed a semistructured script with key questions designed to explore participants’ views on the design of the proposed NoPain Games (Multimedia Appendix 1). A 90-minute timeframe was allowed for in-depth discussions and thorough exploration of the main topics.

#### Feedback Session

At the feedback session, after the playing experience and discussion, participants completed a System Usability Score (SUS) questionnaire [30,31], and verbally provided their feedback on refinements for improving game experience and accessibility. SUS comprises 10 statements, each evaluated on a 5-point Likert scale ranging from “Strongly Disagree” to “Strongly Agree.” It serves as a standardized tool for assessing user experience and pinpointing usability challenges. Widely adopted across academic and industry settings, the SUS has been validated as a reliable benchmark for interface evaluation [32]. Final scores range from 0 to 100, with values above 68 and above 80.3 generally indicating acceptable and excellent usability, respectively [32].

Table 1 tabulates the Implementation Matrix of the study. In the latter, qualitative and quantitative data sources are systematically organized by type, timing of data collection, participant group, and associated research aims. The matrix also outlines anticipated outcomes for each data stream, facilitating transparency in methodological integration and supporting the interpretive logic of the mixed methods design.

**Table 1.** Implementation matrix: overview of qualitative and quantitative data sources, collection timelines, participants, and study aim.

Data source	Type of data	Timing of collection	Participants	Study aim or research question	Anticipated outcome
Expert cocreation session	Qualitative	October 2023	14 experts (6 rheumatologists, 4 researchers, 4 technical experts)	What therapeutic priorities and design requirements should guide the development of NoPain Games?	Identification of core themes: therapeutic benefits, game difficulty, accessibility
Transcribed session dialogs	Qualitative	Postsession (Oct 2023)	Same as above	How do experts perceive the cognitive and physical needs of patients with PsA^a^ in game design?	Thematic analysis yielding subthemes across cognitive and emotional engagement, gameplay adaptability, and inclusive interaction
Patient usability feedback session	Quantitative and qualitative	May 2025	5 patients with PsA (Portugal, the United Kingdom, Netherlands, Greece)	How usable and engaging are the NoPain Game prototypes from a patient perspective?	SUS^b^ score, feedback on game mechanics, difficulty, and interaction design
SUS	Quantitative	May 2025	Same as above	What is the perceived usability of the NoPain Games?	Average SUS score >68
Patient feedback discussion	Qualitative	May 2025	Same as above	What refinements do patients suggest for improving game experience and accessibility?	Insights into difficulty adjustment, technical refinements, and inclusive interaction

a PsA: psoriatic arthritis.

b SUS: System Usability Scale.

### Data Analysis

#### Qualitative Analysis

The qualitative data analysis process used an inductive thematic approach, with 2 independent researchers (first and last authors) analyzing all qualitative data. After thoroughly familiarizing themselves with the data, the researchers developed an initial set of codes, which were subsequently organized into themes and subthemes. These were carefully reviewed to ensure alignment with the data. The final themes and subthemes were crafted using clear and precise language, adhering to the methodology established by Braun and Clarke [33]. To enhance credibility and reliability, the themes underwent cross-validation, and agreement on code assignment was assessed using the Kappa statistic [34], with a threshold of 0.80 deemed acceptable [34]. The finalized themes were determined through consensus-building among the researchers. This approach provided a comprehensive understanding of the participants’ perspectives, ensuring the analysis accurately captured their experiences and insights.

#### Quantitative Analysis

Quantitative data from the SUS questionnaire were analyzed with descriptive statistics, that is, mean, SD, median, IQR, showing how data spread out the central values, and 95% CIs quantifying the uncertainty in estimating the mean. Moreover, SUS 2D (average) and 3D (per patient with PsA) radar plots were estimated along with the SUS ICCs. The ICC is a foundational concept in item response theory, that is, a statistical framework used to model how individuals respond to test items based on an underlying trait or ability [32]. An ICC is a curve that shows the probability p(θ)that a person with a given level of a latent trait θ (eg, usability perception, ability, attitude) will agree with or correctly answer a specific item. The probability p(θ)is given by a 2-Parameter Logistic (2PL) model [35] as


(1) $$p\left(\theta \right)=\frac{1}{1+e^{-a\left(\theta -b\right)}}$$

where a denotes the discrimination (slope), b the difficulty (threshold), and θ the latent trait value. A steep ICC (high a) means the item is highly sensitive to changes in usability perception, that is, ideal for detecting subtle shifts. A right-shifted ICC (high b) means the item is harder to agree with, that is, it activates only at higher usability levels. Finally, a flat ICC (low a) suggests the item does not discriminate well, that is, users across trait levels respond similarly. The whole quantitative analysis was carried out in Matlab 2025a (The MathWorks, Natick, USA).

#### Integration of Findings

Integration of qualitative and quantitative findings occurred through a sequential interpretive process that linked expert-derived design insights to patient-centered usability evaluation. Specifically, themes identified during the expert cocreation session—such as the need for cognitive stimulation, stress reduction, and adaptable difficulty—directly informed the development of 2 new game prototypes (Space Oddity and Four Seasons in the Results section). These prototypes operationalized therapeutic priorities and interaction mechanics discussed during the qualitative phase. In the subsequent feedback session, patients with PsA interacted with these games and provided both quantitative usability ratings via the SUS and qualitative feedback through moderated discussion. The SUS scores offered a standardized measure of perceived usability, while the qualitative comments contextualized these scores by highlighting specific design strengths (eg, intuitive controls, calming visuals) and areas for refinement (eg, onboarding clarity, difficulty pacing). Item-level analysis using ICCs further revealed how individual SUS items aligned with patient-reported experiences, enabling cross-validation of usability dimensions such as learnability and confidence (see Results). This integration strategy ensured that the design decisions were not only expert-informed but also empirically validated through patient engagement, reinforcing the iterative and user-centered nature of the development process.

### Ethical Considerations

This study involved 2 distinct data collection sessions conducted under the iPROLEPSIS Horizon Europe project. They involved members of the iPROLEPSIS Consortium participating in the 2 sessions, complying with the iPROLEPSIS-received ethical approval from the Ethics Committee of Erasmus Medical Center, Rotterdam, Netherlands (MEC-2023‐0470). The first session was a cocreation workshop with members of the iPROLEPSIS Consortium, invited in their professional capacity. The second session was a usability feedback study involving patients with PsA. Neither session involved clinical procedures or the collection of sensitive personal health data. Participation in both sessions was entirely voluntary. For the cocreation session, verbal informed consent was obtained prior to participation. For the usability feedback session, informed consent was obtained via an embedded web-based form presented on the introductory page of the SUS questionnaire (Multimedia Appendix 2), which participants reviewed and accepted before proceeding. In both cases, participants were not compensated for their involvement. To ensure privacy and confidentiality across both phases, all audio recordings were transcribed verbatim and subsequently pseudo-anonymized. SUS survey responses were collected anonymously, with no identifying information linked to individual responses. All data were handled in accordance with General Data Protection Regulation–compliant data protection standards. No personally identifiable individuals appear in any images or supplementary materials.

## Results

### Qualitative Analysis

The cocreation session consisted of 14 participants, including 6 health care professionals, 4 technical experts, 3 observers, and one facilitator. The rheumatologists, all female, had a mean age of 36.3 (SD 7.2) years and over 10 years of professional experience, although most reported a basic level of technology literacy. The technical experts, all male, had a mean age of 44.8 (SD 7.9) years, with more than 15 years of experience and advanced-level technology literacy. The researchers, whose mean age was 28.5 (SD 7.0) years, included 3 females, each with over 4 years of experience and advanced-level technology literacy.

### Thematic Analysis

#### Overview

Thematic analysis across all resolution scales, including themes and subthemes, revealed an almost perfect level of agreement between the 2 independent researchers (first and last authors), as indicated by a Kappa statistic of 0.90. In cases of uncertainty, discussions between researchers led to revisions, ultimately achieving 100% consensus across all subthemes. Data saturation was reached during the final stages of the cocreation session, as no new themes or subthemes emerged despite continued participant engagement. The structured yet open-ended nature of the sessions enabled iterative exploration of perspectives, and the consistency of responses across diverse stakeholders indicated that the thematic landscape had been sufficiently mapped. This saturation supports the robustness and completeness of the qualitative findings. From the thematic analysis, 3 primary themes emerged:

- Therapeutic benefits (Theme 1): This theme included subthemes such as pain distraction, memory enhancement, cognitive stimulation, stress reduction, and creative engagement, emphasizing the games’ potential to help patients with PsA manage both mental and physical challenges.
- Game difficulty (Theme 2): This theme centered on subthemes like balancing gameplay duration, complexity, and adaptive difficulty levels to sustain engagement while avoiding fatigue or diminished therapeutic impact.
- Accessibility and interaction (Theme 3): This theme highlighted subthemes addressing physical limitations, optimizing touchscreen usability, and implementing inclusive design to meet the specific needs of patients with PsA.

The key findings for each identified subtheme are summarized below.

#### Theme 1: Therapeutic Benefits

##### Overview

The therapeutic value of NoPain Games was a recurring topic during the discussions, underlining their role in addressing both physical and mental health challenges. Overall, the NoPain Games were admired not only for their ability to distract patients from pain but also for enhancing cognitive functions like memory. This dual benefit positions them as a promising digital intervention, especially for patients with PsA, who often face fatigue and mental health issues.

##### Subtheme 1: Cognitive Stimulation, Stress Reduction, and Pain Distraction

Specifically, participants emphasized the dual benefits of NoPain Games in providing cognitive stimulation and serving as effective pain distractors. Engaging in memory-focused activities not only addresses the psychological burden of chronic illnesses but also shifts focus away from physical discomfort, ultimately promoting holistic well-being. In particular, the unique appeal of the “Lightning” NoPain Game exemplifies these combined benefits, as one clinician noted:

*I’m just thinking the ‘Lightning’ game is really interesting in the way that besides distracting, it’s also a memory game […] because our patients are depressed, and this can be very helpful.*

[Rheumatologist #2]

This sentiment highlights the potential of such games to uplift patients’ moods and combat depression, showing the therapeutic value of cognitive engagement. Some participants further reinforced this aspect by stating,

*I like this game [the ‘Lightning’ game], it promotes memory skills, which can be seen as pain distractors.*

[Rheumatologist #3]

*[…] the underlying mechanisms for this game [The Garden], is the memory, the mental stimulation in a way as part of the pain distractor.*

[Researcher #2]

By promoting cognitive stimulation, these serious games can also contribute indirectly to pain management, reducing the focus on discomfort. Additionally, many participants acknowledged these games as powerful tools for pain distraction and stress reduction. One participant summarized this aspect succinctly, as follows:

*The NoPain Games are intended to act as a pain distractor, allowing the patient to focus on it as a means of distraction, which in turn can support pain management and stress reduction.*

[Researcher #1]

Overall, NoPain Games intends to integrate calming experiences and mentally stimulating activities, offering therapeutic benefits that extend beyond traditional treatments. By providing a distraction from chronic illness discomfort, these games shift patients’ focus to engaging tasks that can alleviate stress, improve mood, and foster a sense of achievement. Their holistic approach addresses cognitive, emotional, and physical well-being that can enhance therapy outcomes and empower patients with PsA to actively participate in their care journey.

##### Subtheme 2: Promoting Creativity

This subtheme explores the integration of creativity into gameplay as a strategy for enhancing both physical and mental engagement in therapeutic contexts. Incorporating creative elements, such as drawing flowers in The Garden game, can introduce a unique combination of movement and creativity, directly supporting therapeutic objectives. As highlighted by one clinician:

*Incorporating creative elements, such as drawing flowers, combines movement with creativity, stimulating the mind and physical tasks. Maybe the patient could draw the flower in this game [The Garden]. So you can combine in this way the movement and the creativity.*

[Rheumatologist #6]

Furthermore, another clinician proposed expanding this creative aspect, as follows:

*[…] maybe there is a stage where somehow someone can use this kind of flowers to build something […] more constructive.*

[Rheumatologist #5]

Incorporating creative tasks into gameplay can potentially enhance interaction and personalization, encouraging active patient engagement in rehabilitation. Activities like drawing or constructing can stimulate fine motor skills, hand-eye coordination, cognitive focus, and emotional well-being. This creative emphasis can expand the NoPain Games’ role, making them valuable tools that complement traditional treatments while combining therapeutic movement with self-expression for a more meaningful and enjoyable therapeutic experience.

### Theme 2: Game Difficulty

#### Overview

Balancing the duration and complexity of gameplay emerged as a crucial aspect of game design during the cocreation session. Clinicians expressed concern that both extremes—prolonged sessions and overly simplistic mechanics—could undermine the games’ therapeutic and engagement potential.

#### Subtheme 1: Managing Fatigue and Engagement

Prolonged gameplay or repetitive motions might exacerbate fatigue, reducing the games’ effectiveness as therapeutic tools. One clinician pointed out the following:

*For instance, it could be interesting to know how long the game sequences of the game [The ‘Lightning’ game] are, so how long should it last.*

[Rheumatologist #5]

This indicates the need for carefully tailored session lengths to prevent overstimulation. On the other hand, overly simple game mechanics might fail to captivate players or provide meaningful stimulation. In this line, one clinician also highlighted this issue, noting:

*So, I'm just thinking about the complexity of the game [The ‘Lightning’ game], in particular the duration of the sequence and the number of repetitions to play the game. […] have you decided or thoughts regarding the game duration?*

[Rheumatologist #1]

This perspective highlights the need for future work to focus on designing gameplay that achieves the optimal balance—sufficiently challenging to maintain engagement, yet not so demanding as to provoke frustration or exhaustion.

#### Subtheme 2: Customizable Difficulty Levels

This subtheme introduces customizable difficulty levels and adjustable session durations as effective solutions. These features enable players to adapt gameplay to their individual needs, thereby enhancing both satisfaction and therapeutic outcomes. Furthermore, the significance of gradual progression in difficulty is emphasized by one participant:

*For each level of difficulty, different coordination movements can be incorporated to offer a light yet progressively challenging experience, possibly linked to timing.*

[Researcher #1]

This highlights how incorporating varied coordination movements fosters engagement while simultaneously supporting therapeutic goals, such as enhancing refined motor skills and cognitive reaction time. By offering challenges that escalate at an adaptable pace, players can feel a sense of growth and accomplishment, reinforcing motivation and adherence to therapeutic activities. Additionally, incorporating a time-based element was suggested as a potential customization option. As noted by one technical expert:

*Can I also ask if the game [The ‘Lightning’ game] is connected to time? Does it involve reaction time that progressively get faster? For example, if there’s a time limit, would it require completing the task within 5 seconds, then 10 seconds?*

[Technical expert #1]

Together, these insights can suggest that customization in difficulty and session dynamics is vital for creating a flexible and impactful game experience.

### Theme 3: Accessibility and Interaction

#### Overview

Accessibility was identified as another pivotal theme, reflecting the need for an accessible design to accommodate the physical limitations of patients with PsA. Symptoms like joint stiffness, swelling, and reduced dexterity can significantly impact interaction with digital tools, including games.

#### Subtheme 1: Physical Limitations and Interface Challenges

Overall, many clinicians highlighted specific challenges related to physical symptoms. For example, one clinician commented:

*I guess if people have swollen fingers, the accuracy for this game [‘The Garden’ game] might be a problem, in the sense that you're trying to get quite a small area when you've got lots of flowers like this and the accuracy is potentially trickier.*

[Rheumatologist #6]

This underlines the need for larger touch targets and simplified game interactions to accommodate patients with swollen fingers or a limited range of motion. Similarly, another clinician noted:

*Sometimes, on a small screen, it’s quite difficult to drag a marker to a particular point or to be very accurate with where we're pressing if we've got bad hand arthritis.*

[Rheumatologist #2]

This observation points to the importance of designing user interfaces (UIs) that minimize the need for precise movements, making the games more accessible to those with severe joint issues.

#### Subtheme 2: Enhancing Inclusivity

To make the games more inclusive, developers could implement features such as adjustable touch sensitivity, alternative input methods (eg, voice commands), and options to magnify specific screen elements. These adaptations would enhance usability and ensure that the games can be enjoyed by a wider range of patients, regardless of their physical limitations.

These features foster a more inclusive experience for users with diverse needs, particularly those with conditions impairing their motor skills or visual acuity. For instance, adjustable touch sensitivity would allow users with reduced dexterity to interact with the interface more comfortably, while alternative input methods, such as voice commands, could provide a hands-free option for individuals unable to perform precise touch gestures. Similarly, magnification options for specific screen game elements could support users with visual impairments, ensuring they can navigate the game environment with ease and confidence. As suggested by one participant:

*In fact, the levels of difficulty and synchronization with the user profile, as suggested before using the adaptation algorithm of the games (…) includes the patient’s disease status, and eventually align with the hand or finger exhibiting better conditions in terms of symmetry and functionality.*

[Researcher #1]

This highlights the potential for an adaptive system that tailors the gameplay experience to the specific physical conditions of the player. By considering factors such as hand symmetry, finger functionality, or disease progression, the game could dynamically adjust its mechanics to align with each patient’s capabilities. For example, if a player has difficulty using one hand due to PsA, the game could prioritize interactions that favor the less-affected hand or allow for single-hand play. This ensures an engaging experience, minimizes frustration, and enhances therapeutic outcomes. Furthermore, a robust adaptation algorithm could learn and evolve with the player’s needs, offering more personalized solutions over time by focusing on inclusive design principles.

Summarizing, these detailed insights into each theme emphasize the multifaceted nature of NoPain Games’ design and impact. By addressing therapeutic benefits, gameplay balance, and accessibility, these games have the potential to make a meaningful difference in the lives of patients with PsA. Although the feedback from this session highlighted the clinical value of the storyboard elements and provided suggestions for design enhancements, there remains an opportunity to develop prototypes that integrate all these ideas to guide future development. The storyboards were revised based on the feedback collected, and a wider range of game mechanics was incorporated to improve the overall design and gameplay. Consequently, 2 NoPain game prototypes*—*Space Oddity Game and Four Seasons Game—have been conceptualized, as detailed next.

### Prototyping the NoPain Games

#### Overview

**Figure 5.**
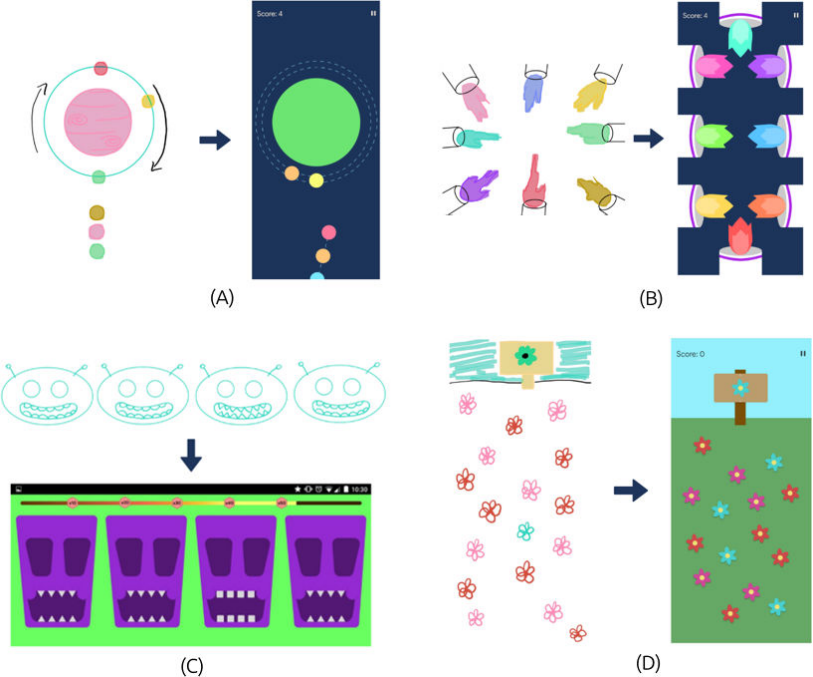
Refined storyboards for the proposed NoPain Games: (A) Space Oddity coordination challenge, (B) Space Oddity memory challenge, (C) Space Oddity visual perception challenge, and (D) Four Seasons.

Building on the subsequent phase of the agile approach after the ideation and storyboard design phase, 2 NoPain Games prototypes for pain relief were developed, incorporating targeted feedback gathered during the cocreation session (Development phase in Figure 1). These prototypes include Space Oddity (Figure 5), a space-themed serious game featuring 3 minigames that engage players in memory, coordination, and visual perception tasks; and Four Seasons (Figure 5D), a nature-themed game designed to promote repetitive, soothing movements. Tables2  3 provide an overview of the key characteristics of the proposed NoPain Game prototypes, including their game concepts, clinical value, visual design, game mechanics, and difficulty progression.

**Table 2.** Main characteristics of the proposed Space Oddity game prototypes, featuring 3 distinct mini-game–related areas, that is, memory-based challenge, coordination skills, and visual perception, each designed to target specific cognitive and motor functions associated with psoriatic arthritis. For each area, the game concept, intended clinical value, visual design elements, core gameplay mechanics, and difficulty progression are described.

	Memory-based challenge	Coordination skills	Visual perception
Game concept	Players memorize and reproduce a sequence of ignited spaceships.	Players launch asteroids into orbit while avoiding collisions.	Players identify the odd creature among a group.
Clinical value	Strengthening cognitive and memory sequencing skills.	Developing motor coordination by improving reaction timing and precision.	Improving focus, visual perception, and cognitive processing speed.
Visual design	Embracing the game’s space esthetic, the spaceships’ ignition includes vibrant and distinct hue colors to strengthen visual memory cues during gameplay, with clear contrast against the background for easy recognition.	A central, colorful planet with orbiting asteroids stands out against a calm-toned universe, with new asteroids emerging from below to ensure an intuitive and structured gameplay flow.	Aligned with the futuristic theme, alien robot-like creatures were chosen as the main characters, since various features (such as eye color, teeth shape, and accessories) can be easily modified for a dynamic experience.
Game mechanics	At the start of each round, a sequence of rockets lights up. The player must replicate the sequence by tapping the rockets in the correct order. The game verifies the input, granting progression if correct. If incorrect, the sequence repeats for the player to try again.	The game consists of multiple stages, each with a set number of asteroids. Players must launch asteroids into orbit while ensuring they do not collide. When all asteroids are placed successfully, a new planet appears. If 2 asteroids collide, the game ends.	A set of creatures is displayed, with one differing in subtle ways. The player must identify the intruder. Feedback is given through color and audio cues.
Difficulty progression	The sequence length increases as the game progresses, making it more challenging. An easier mode maintains the sequence and adds only one new element per round, while a harder mode randomizes the sequence each time, increasing difficulty.	Initially, asteroids move in a straight path toward the orbit. At higher difficulty levels, additional asteroids move dynamically, requiring more precise timing. Some areas on the planet may be restricted (eg, mountains), and color-coded zones may require matching asteroids.	At lower difficulties, the creatures’ differences are more apparent. At higher levels, the distinctions become more subtle, requiring greater attention to detail.

**Table 3.** Main characteristics of the proposed Four Seasons Game prototypes, targeting 2 distinct areas, that is, visual perception and coordination skills, each designed to support stress reduction, focus, and motor precision in individuals with psoriatic arthritis. For each area, the game concept, intended clinical value, visual design elements, core gameplay mechanics, and difficulty progression are described.

	Visual perception	Coordination skills
Game concept	Players identify specific flower species on a garden field.	The player has to select fruits to fall from a tree, while ensuring they fall into a basket and not on the floor.
Clinical value	Stress reduction, improved focus, and coordination.	Reaction time and precision.
Visual design	A relaxing nature-based theme in the form of a garden. Various colorful plants and flowers populate the garden, giving it a vibrant atmosphere.	A nature-based theme with fruit trees in the background full of colorful fruits.
Game mechanics	The player is instructed to find and tap a chosen flower species, in a garden scenario full of various plants. Feedback is provided to let the player know if they selected the correct flower. As the game progresses, the seasons change, creating a dynamic environment and increasing the level of challenge.	The player has to select fruits to fall from a tree, as a rolling bucket passes underneath. The player gains points if they correctly calculate the timing of the falling fruit, making them land in the basket. They lose points if the fruits miss the basket instead.
Difficulty progression	As the game progresses, some elements become more challenging. For example, the flowers are no longer static; they start moving or become more similar to each other.	As difficulty rises, the number of fruits increases, the basket moves faster, and the basket becomes smaller.

#### Space Oddity

The Space Oddity NoPain Game was inspired by the space theme expressed in Figure 3B and incorporated a memory-based challenge with a patterns-matching mechanic similar to that in Figure 3A. Based on clinician feedback provided at the second design phase, other game tasks related to coordination and visual perception were added, while maintaining the overall space thematic and base mechanics. Table 2 links the specific game tasks with the themes highlighted at the second cocreation session. Throughout the game, players should experience all these tasks in the form of minigames. Therefore, as the players enter Space Oddity and select a difficulty level, 3 minigames appear in a different order to avoid monotony and ensure that the game will be played various times without getting repetitive.

The first minigame is designed to train coordination skills (Figure 5A). A main planet stands at the center of the screen, while being surrounded by a fixed-speed orbit of asteroids. As new asteroids emerge from below, the player/patient must launch them into orbit one at a time, ensuring they do not collide with existing asteroids. Success depends on precise timing, requiring the player/patient to tap the screen at the right moments to avoid collisions. After a certain number of asteroids are launched, a new planet emerges with new challenges. Additional features to add diversity to the game include areas on the planet with mountains where asteroids cannot be placed, or color-coded zones to correspond to asteroids of the same color (Figure 5A). The second minigame, depicted in Figure 5B, features 8 spaceships scattered across the screen, all starting with their engines turned off. It operates, like the Simon game [36], in rounds where players watch as a sequence of rockets ignite and then are required to replicate the sequence by selecting the rockets in the correct order. The first round begins with a single randomly ignited rocket. In each subsequent round, the previous sequence repeats in the same order, with one additional rocket added. The minigame continues until a certain number of rounds is successfully completed. To enhance memorization, unique colors and musical notes can be attributed to each rocket, providing both visual and auditory stimuli. Figure 5B illustrates the core prototype elements and an example screen in which all rockets are ignited with their distinct colors. The third minigame focuses on memory, concentration, and visual perception, where a player/patient performs several rounds of identifying from a set of 4 creatures, the one that differs from the others (intruder) (Figure 5C). Overall, various features may be modified to create distinctions, such as the creatures’ shape or variations in their elements.

#### Four Seasons

The Four Seasons NoPain Game builds upon the concepts illustrated in the storyboard of Figure 3C. Similarly, Tables2  3 link the specific game tasks with the themes highlighted at the second cocreation session. During gameplay, the players/patients explore a garden filled with various flowers and are asked to identify a specific one (Figure 5D). As the game progresses, the seasons change, affecting both the environment’s aesthetic and the challenges presented. Each season introduces unique difficulties and advantages, adding variety to the game. The Four Seasons game was designed to distract players from pain by offering 2 distinct modes to suit individual preferences. The Relax Mode allows players to peacefully search for flowers in a garden across different seasons, while the Party Mode introduces added excitement with minigames at the end of each season for a more dynamic experience. Overall, this game, designed to reflect the unique characteristics of each season, has the potential to offer a refreshing gameplay variety while introducing an element of dynamism for players/patients seeking a more immersive experience.

### Feedback From Patients With PsA

#### Quantitative Analysis

The patients with PsA feedback group included 2 women and 3 men, with ages ranging from 25 to 64 years, split into age groups of 25‐34 years (1), 35‐44 years (1), 45‐54 years (2), and 55‐64 years (1). All patients had been diagnosed with PsA for more than 8 years; had an average experience in gaming, and good experience in smartphone usage. During the session, 4 participants (1-4) reported experiencing a PsA flare.

The session first focused on “Space Oddity” through a playable version developed in Unity3D (Figure 4A). Participants played the game and then evaluated its usability by completing the SUS questionnaire. Table 4 tabulates the derived descriptives from the SUS data, that is, mean (SD), 95% CI, and median (IQR). From the latter, it is seen that an average SUS score of 79 (SD 10.4; 95% CI 69.89‐88.11) was reached, suggesting good overall usability, with some variation in individual user perceptions.

**Table 4.** Descriptives of the System Usability Scale (SUS).

Item definitions	Mean (SD)	95% CI	Median (IQR)
Frequency Use (1)	3.80 (0.84)	3.07‐4.53	4 (3-4)
Complexity (2)	2.40 (1.95)	0.69‐4.11	1 (1-4)
Ease of Use (3)	4.60 (0.55)	4.12‐5.08	5 (4-5)
Support Needs (4)	1.00 (0.0)	1.00‐1.00	1 (1-1)
Functionality Integration (5)	3.80 (0.84)	3.07‐4.53	4 (3-4)
Inconsistency (6)	2.00 (1.0)	1.12‐2.88	2 (1-3)
Confidence (7)	4.40 (0.55)	3.92‐4.88	4 (4-5)
Cumbersomeness (8)	1.60 (0.90)	0.82‐2.38	1 (1-2)
Learnability (9)	4.20 (0.84)	3.47‐4.93	4 (4-5)
Learning Curve (10)	2.20 (1.64)	0.76‐3.64	2 (1-2)
Total SUS	79 (10.4)	69.89‐88.11	75 (75‐82.5)

**Figure 6.**
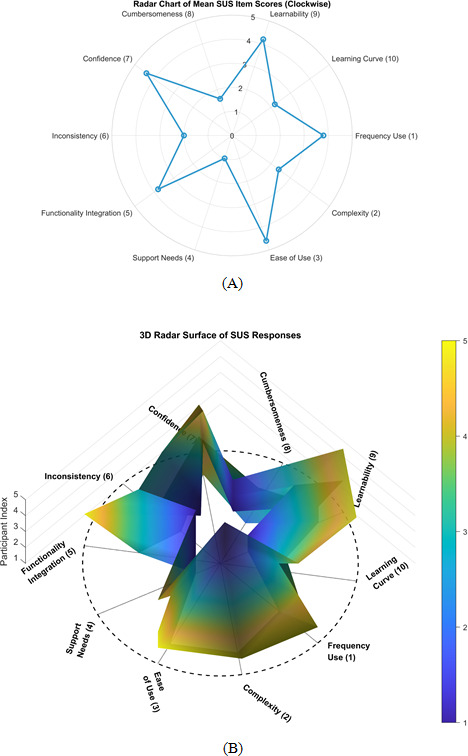
(A) Radar chart of mean SUS item scores arranged clockwise, highlighting usability strengths and weaknesses. (B) 3D radar surface plot of individual SUS responses, showing participant-level variation across usability dimensions. SUS: System Usability Scale.

Moreover, Figure 6 displays a 2D radar plot of average SUS responses across all participants, with each axis representing a usability factor mapped to its corresponding SUS item number (eg, Frequency Use (1), Cumbersomeness (8), etc). The radial layout allows for intuitive comparison of agreement levels across items, with values ranging from 1 (“Strongly Disagree”) to 5 (“Strongly Agree”). Peaks in the plot are observed around positively worded items such as Ease of Use (3), Confidence (7), and Learnability (9), indicating strong agreement and direct contributions to higher SUS scores. Conversely, troughs appear around negatively worded items like Support Needs (4), Cumbersomeness (8), and Learning Curve (10)*,* where low agreement is desirable and reflects positive usability perceptions. The overall shape of the radar plot reveals a balanced usability profile, with high agreement on core interaction elements and consistent disagreement with statements implying complexity or poor integration.

In addition, Figure 6B presents a 3D radar surface plot where axes radiate outward from the center, each labeled with a usability factor and its corresponding SUS item number, for example, Consistency (6) and Learning Curve (10). The surface height represents participant index, while the color gradient (blue to yellow) encodes response values from 1 to 5. The plot reveals structural patterns in usability perception, with elevated regions around Ease of Use (3), Confidence (7), and Learnability (9), positively worded items where high agreement directly contributes to higher SUS scores. Conversely, lower scores on negatively worded items such as Complexity (2), Support Needs (4), Inconsistency (6), Cumbersomeness (8), and Learning Curve (10) are desirable, as they reflect disagreement with statements implying poor usability. Notably, Support Needs (4) received a uniform score of 1 across all participants, indicating unanimous disagreement with the notion that external support was required to use the system, an encouraging signal of intuitive design and self-sufficiency. Participant-level differences are also evident. In particular, Participants 1‐4, who reported experiencing PsA flare episodes during evaluation, showed slightly more variability in items such as Functionality Integration *(5*) and Inconsistency *(6*), suggesting that symptom severity may influence perceptions of system responsiveness and coherence. In contrast, Participant 5, who did not report a flare, exhibited consistently high agreement on positively worded items and low agreement on negatively worded ones, resulting in a smoother and more elevated usability profile across the radar surface. Together, Figure 6A and B offer complementary perspectives on usability perception, highlighting both structural consistency and individual-level nuances.

**Figure 7.**
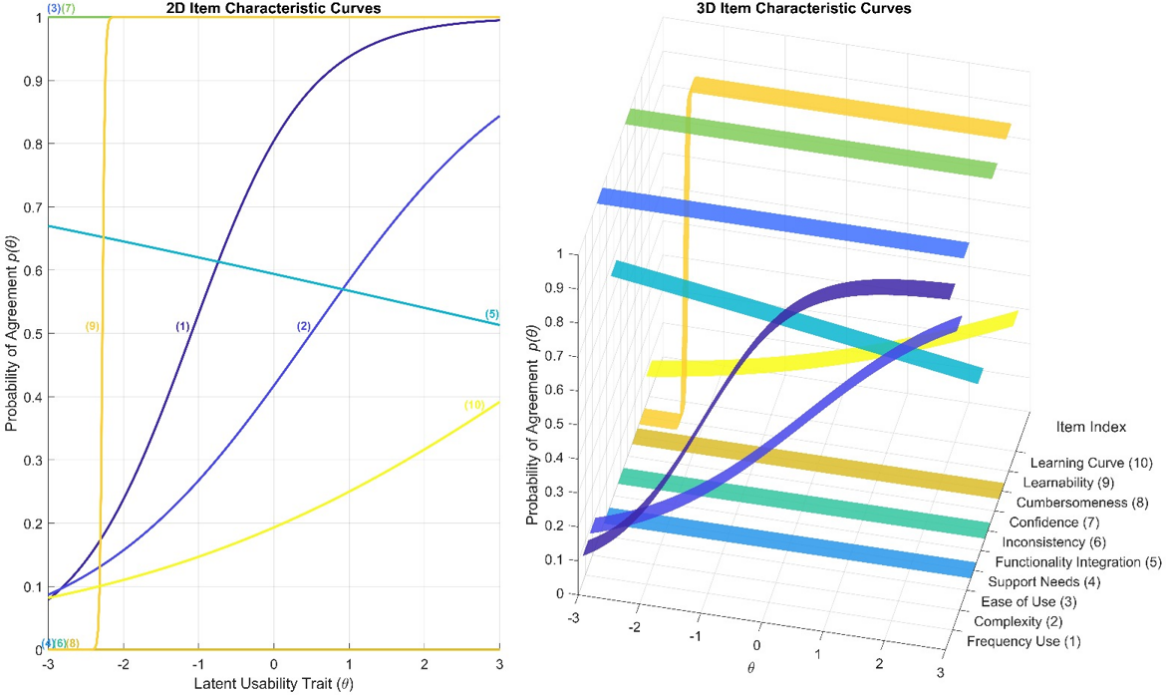
2D Item characteristic curves (left) showing agreement probability p(θ) across latent usability trait (θ), with each curve labeled by item index, along with the 3D item characteristic curve ribbons (right) for all System Usability Scale items, visualizing response probabilities across θ and item index, with full item labels on the horizontal axis.

Furthermore, Figure 7 illustrates a detailed visualization of item-level behavior across the latent usability trait spectrum. The left panel presents 2D ICCs for selected SUS items, each labeled by item index. These curves illustrate the probability of agreement p(θ) (see (1)) as a function of latent usability (θ), revealing distinct patterns of discrimination and difficulty. The right panel complements this view with a 3D ICC ribbon plot, where each SUS item is vertically separated and labeled by its full descriptor. This format enhances interpretability by exposing how response probabilities vary not only across θ but also across items. Moreover, Table 5 includes the corresponding parameters (a,b) (see (1)) of the ICCs depicted in Figure 7. Analytically, from Figure 7 and Table 5, the following observations can be derived per SUS item, revealing their heterogeneity:

- Frequency of Use (1)-positive wording: With a = 1.29 and b = –1.10, this item shows moderate discrimination and early activation. The curve is gently sloped and elevated across the trait spectrum, indicating consistent agreement even at low usability levels. This suggests it reflects habitual or contextual familiarity rather than sensitivity to usability changes. Its contribution is stable but diagnostically limited.
- Complexity (2)-negative wording: With a = 0.67 and b = 0.49, the curve rises gradually and activates in the mid-range of θ. Users increasingly disagree as usability improves, aligning with expected polarity. However, the low a value indicates weak discrimination. The ribbon is broad and shallow, offering general friction-related information but lacking precision.
- Ease of Use (3)-positive wording: Despite its conceptual importance, a = 1.73×10⁻¹³ and b = –5.93×10¹⁴ suggest a modeling anomaly, likely due to uniform agreement across participants. The curve appears flat and saturated, with no meaningful slope. While users universally endorse this item, its statistical discrimination is negligible, making it confirmatory rather than diagnostic.
- Support Needs (4)-negative wording: With a = –8.72×10⁻¹⁴ and b = –1.18×10¹⁵, this item exhibits modeling instability. The curve is flat and extremely right-shifted, indicating that nearly all users disagreed regardless of usability level. In fact, all users disagreed. This uniform rejection aligns with high usability but limits the item’s ability to differentiate experiences. It may be conceptually relevant but statistically redundant.
- Functionality Integration (5)-positive wording: With a = –0.11 and b = 3.48, the curve is shallow. The negative a suggests an inverse slope, possibly due to inconsistent response patterns. The ribbon is low and delayed, indicating poor discrimination and limited responsiveness.
- Inconsistency (6)-negative wording: Like item 4, a = –8.72×10⁻¹⁴ and b = –1.18×10¹⁵ reflect modeling collapse. The curve is flat and nondiscriminative, with uniform disagreement across users. While this aligns with high usability, the lack of slope or variability renders it statistically inert.
- Confidence (7)-positive wording: With a = 1.73×10⁻¹³ and b = –5.93×10¹⁴, this item shows extreme early activation and saturation. The curve is flat and elevated, indicating universal agreement. Like *Ease of Use (3*), it functions as a strong confirmatory item but lacks statistical discrimination due to the ceiling effect.
- Cumbersomeness (8)-negative wording: Again, a = –8.72×10⁻¹⁴ and b = –1.18×10¹⁵ suggest modeling failure. The curve is flat and low, with near-universal disagreement. While this reflects low friction, the lack of variability limits its diagnostic value.
- Learnability (9)-positive wording: With a = 59.00 and b = –2.28, this item shows extremely high discrimination and early activation. The curve is steep and sharply contoured, making it the most responsive item in the set. The ribbon is narrow and elevated, indicating strong sensitivity to usability perception across the trait range. This item is statistically and conceptually robust.
- Learning Curve (10)-negative wording: With a = 0.33 and b = 4.33, this item exhibits low discrimination but great difficulty. The curve rises gradually and activates only at the upper end of the usability trait spectrum. The ribbon in Figure 7 is delayed but upward-sloping, confirming that the item captures late-stage usability clarity, particularly in systems that excel in onboarding. While its responsiveness is limited at lower θ levels, it still contributes meaningful coverage of the high-usability tail, making it useful for identifying systems with strong learnability.

From the aforementioned, we can identify four interpretive categories: (1) Statistically robust item: “Learnability (9)” shows exceptional discrimination and early activation, making it the most diagnostically powerful item. (2) Confirmatory but saturated items: ”Ease of Use (3)” and “Confidence (7)” are universally endorsed but exhibit near-zero discrimination due to ceiling effects. They confirm high usability but offer limited differentiation. (3) Low-performing or inert items: “Support Needs (4),” “Inconsistency (6),” and “Cumbersomeness (8)” show flat, nonresponsive curves and modeling collapse, limiting their diagnostic utility. (4) Mid-range and coverage items: “Frequency of Use (1),” “Complexity (2),” and “Learning Curve (10)” contribute moderate or delayed responsiveness, offering coverage across the trait spectrum but with limited precision. This item-level interpretation supports evaluation of the SUS, guiding decisions on item weighting and further scrutiny for potential refinement.

**Table 5.** Estimated parameters (a, b) of the corresponding item characteristic curves of Figure 7 for each System Usability Scale (SUS) item.

SUS item	Alpha (a)	Beta (b)
Frequency Use (1)	1.29	–1.10
Complexity (2)	0.67	0.49
Ease of Use (3)	1.73×10^–13^	–5.93×10^14^
Support Needs (4)	–8.72×10^–14^	–1.18×10^15^
Functionality Integration (5)	–0.11	3.48
Inconsistency (6)	–8.72×10^–14^	–1.18×10^15^
Confidence (7)	1.73×10^–13^	–5.93×10^14^
Cumbersomeness (8)	–8.72×10^–14^	–1.18×10^15^
Learnability (9)	59.00	–2.28
Learning Curve (10)	0.33	4.33

#### Qualitative Analysis

During the patients with PsA feedback session, the group was guided through a design prototype demonstration of the Four Seasons (Figure 4B), covering its tutorial, practice session, navigation, gameplay, bonus level, and scoring. Feedback was generally positive and constructive, with discussions focusing on several key areas across the 2 games. In particular, they proposed:

- resolving iOS compatibility issues to improve accessibility,
- modifying difficulty levels and incorporating adaptive artificial intelligence (AI) in “Space Oddity” to tailor challenges to individual player profiles,
- transitioning “The Four Seasons” from prototype to full interactive development with seasonal mechanics and high-quality 3D rendering, and
- introducing smartwatch integration to track stress levels before and after gameplay in both games.

From a combined perspective, the derived average SUS score of 79 reflects a generally positive user experience, placing the NoPain Games within the “good” usability range according to established benchmarks. This score suggests that core interaction elements, such as navigation, responsiveness, and interface clarity, were well-received across a diverse group of patients with PsA, even during flare episodes. To better understand how this overall score reflects item-level behavior, we examined the individual ICC profiles. The accompanying ICC analysis revealed several positive outcomes. These item-level patterns help contextualize the overall SUS score of the NoPain Games, revealing how specific usability dimensions contributed to the positive user experience. This is combined with the feedback provided during the session, which revealed that usability alone does not fully capture user expectations in therapeutic contexts. Participants articulated a clear desire for functional enhancements and personalization features that extend beyond baseline usability. Their suggestions, ranging from adaptive AI and platform compatibility to immersive rendering and biometric integration, indicate that patients are not merely evaluating ease of use, but actively envisioning how the system could evolve into a more responsive and clinically meaningful tool. This feedback reveals the importance of interpreting usability scores in tandem with qualitative insights, especially when designing interventions for chronic conditions where therapeutic relevance and emotional engagement are critical.

## Discussion

### Main Findings

This study aimed to explore expert perspectives on the design of 2 serious games, that is, NoPain Games, intended to alleviate chronic pain and improve quality of life for individuals with PsA. The cocreation session revealed 3 key thematic areas: therapeutic benefits, game difficulty, and accessibility and interaction. These themes directly informed modifications to the game prototypes, aligning with the study’s objective to develop personalized, user-centered digital interventions for chronic pain management. Moreover, the usability assessments of patients with PsA using SUS and feedback further supported the design approach, indicating generally positive perceptions of the game prototypes and reinforcing their potential as accessible and engaging tools for digital pain management. The extended quantitative analysis of SUS responses, including item-level discrimination and difficulty modeling via ICCs, provided deeper insight into usability perceptions. These findings offer item-level insights that can guide targeted enhancements to interface design and interaction mechanics. This quantitative granularity complements the thematic findings, particularly those related to therapeutic intent and user engagement.

Therapeutic benefits were a major focus, with the games designed to provide pain distraction and cognitive engagement. For example, the “Lightning” game focuses on memory enhancement, which can help distract from pain and improve cognitive function [3]. Additionally, the games incorporate soothing and repetitive mechanics, such as in the “Flashing Stars” game, which promotes relaxation and stress reduction [6]. Moreover, previous studies had also suggested a link between serious games and improvements in working memory [37], prompting further research on this topic.

Game difficulty was another critical theme, with feedback emphasizing the need to balance game duration and complexity to maintain engagement without causing fatigue. This led to the development of adaptive difficulty levels that adjust to the player’s abilities [38]. The games were designed to be challenging enough to keep players engaged but not so difficult that they become frustrating. This balance is crucial for maintaining the therapeutic benefits of the games [39]. In fact, the challenge of balancing game difficulty has been identified in previous studies [38,39], yet it remains complex due to the need to provide therapeutic benefits while maintaining player engagement. When designing serious games, it is essential to balance activity levels, as over-exercise can cause fatigue and frustration, while insufficient activity may reduce therapeutic benefits. Adaptive difficulty levels aligning with players’ abilities and goals could ensure engagement, gradual improvement, and overall well-being.

Accessibility and interaction were also key considerations. Overall, the game design considered the physical limitations of patients with PsA, such as joint stiffness and swelling. This led to the optimization of touchscreen usability and the inclusion of larger touch targets to accommodate swollen fingers [40]. Ensuring that the games are accessible to all patients with PsA, regardless of their physical abilities, was a key focus. This included designing intuitive and easy-to-use interfaces [41]. Moreover, smartphone accessibility has been analyzed in various domains [40,41] due to the fine motor skills required for small touchscreen interactions. However, its impact on patients with PsA could be investigated further.

The alterations in game design during the cocreation workshops align with findings from existing literature on serious games and digital health interventions. Studies have shown that serious games can reduce stress, enhance cognitive function, and provide pain relief through immersive and engaging experiences [3,6]. The NoPain Games leverage these benefits by incorporating memory and relaxation activities. The importance of balancing game difficulty to sustain engagement and therapeutic efficacy is well-documented. Adaptive difficulty levels, planned for implementation in the NoPain Games, are crucial for maintaining player interest and ensuring the games’ effectiveness [38,39]. Research highlights the need for accessible game design, especially for individuals with physical limitations. The NoPain Games’ focus on optimizing touchscreen usability and inclusive design reflects these findings [40,41]. Furthermore, the conceptual framework (2D-ME) for explaining self-first and self-third person views of prototyping dynamics in serious games design highlights the importance of iterative feedback and dynamic constructs in the game design process [42]. This framework supports the iterative refinement seen in the NoPain Games development, ensuring that the games are both engaging and therapeutic.

Furthermore, the design of NoPain Games leverages key insights into how the human brain pays attention to various aspects of game design. By understanding and applying principles related to perception, memory, attention, and emotional engagement, the developers can create games that are both therapeutic and engaging.

For example, perception plays a crucial role in how players interact with NoPain Games. The brain processes visual and auditory information to understand and navigate the game world. For instance, the “Lightning” NoPain game uses effective visual cues and sound effects to guide players’ attention and enhance their immersion [43]. The UI design is also critical, ensuring that players can easily access information and controls without being distracted by confusing or cluttered interfaces. This is particularly important for patients with PsA, who may have physical limitations affecting their game interaction [40].

Memory is another essential aspect of the proposed NoPain Games. Players rely on short-term memory to remember recent actions, objectives, and game mechanics. This is particularly important in fast-paced games where quick decision-making is required. For example, the “Lightning” NoPain Game focuses on memory enhancement, helping players to distract from pain and improve cognitive function [3]. Long-term memory is engaged when players learn and remember game rules, storylines, and strategies. By reinforcing learning through repetition and rewards, NoPain Games can enhance player retention and engagement [43].

Attention is also vital for maintaining focus and engagement in the proposed NoPain Games. The brain’s ability to maintain focus is critical for sustained engagement, and the developers have used various techniques to capture and hold players’ attention. This includes compelling narratives and dynamic gameplay [44]. Managing cognitive load is essential to prevent player fatigue. NoPain Games balance complexity and simplicity to keep players engaged without overwhelming them. This involves designing intuitive controls, clear objectives, and manageable challenges, ensuring that the games are accessible and enjoyable for patients with PsA.

Emotional and motivational factors significantly influence the brain’s attention to NoPain Games. Emotions play a significant role in how players experience the games. Engaging storylines, relatable characters, and emotional rewards enhance player immersion and satisfaction. The brain responds to rewards and achievements, which can motivate players to continue playing. NoPain Games use various reward systems, such as points and progression systems, to keep players motivated and engaged [43].

Achieving a flow state is another critical aspect of NoPain Games. The concept of “flow” refers to a state of deep focus and immersion where players lose track of time [45]. Achieving flow involves balancing challenge and skill, providing clear goals, and offering immediate feedback. NoPain Games successfully induce flow by creating a balance between challenge and skill, ensuring that players remain deeply engaged and enjoy the therapeutic benefits of the games [43].

Approaching the aforementioned from the lens of the 3 themes identified, that is, therapeutic benefits, game difficulty, and accessibility and interaction, a deep interwovenness can be identified with sustained engagement, player attention, cognitive load, player fatigue, emotions, and flow state in the context of NoPain Games for patients with PsA. In particular:

1. Therapeutic benefits: NoPain Games aim to alleviate pain and improve emotional well-being by providing cognitive stimulation, stress reduction, and memory enhancement. These benefits are crucial for fostering sustained engagement as players find therapeutic value in their activity, which keeps their attention fixed on the games. The soothing and predictable mechanics of games like “Flashing Stars” reduce *cognitive load* by presenting clear objectives and repetitive tasks, allowing players to focus without being overwhelmed. This emotional engagement also helps maintain the ideal flow state, where players are immersed in gameplay and distracted from their chronic pain.
2. Game difficulty: Balancing and adapting difficulty levels directly supports sustained engagement by keeping tasks manageable yet stimulating. Customizable difficulty prevents player fatigue, ensuring sessions remain enjoyable rather than exhausting. A dynamically adjusted challenge ensures that attention is maintained without inducing frustration, helping to optimize both players’ attention and cognitive effort. Gradual progression of difficulty, such as increasing complexity in the “Lightning” game, enhances emotions of achievement and satisfaction, which is key for reinforcing engagement and maintaining the flow state.
3. Accessibility and interaction: The design considerations in this theme, such as larger touch targets and intuitive interfaces, ensure that physical limitations do not hinder participation. This inclusivity supports sustained engagement by enabling patients to interact easily and prevent frustration. These accommodations reduce the cognitive load on players, allowing them to focus on the tasks rather than overcoming interface challenges. This theme also reduces players’ fatigue by minimizing physical strain and frustration, helping to maintain a relaxed state essential for emotional well-being and immersion into a flow state.

Together, these themes create a synergistic experience where accessibility removes barriers, therapeutic benefits uplift emotional and cognitive states, and adaptive difficulty sustains attention and motivation, all of which harmonize to engage patients with PsA while mitigating pain effectively.

The iterative optimization process in developing NoPain Games prototypes involved refining visual cues, simplifying the UI, balancing cognitive load, and incorporating emotional and motivational elements. By understanding how the brain pays attention to different aspects of game design, the designers/developers were able to create games that are both effective and enjoyable for patients with PsA. This approach ensured the potential for the proposed NoPain Games to provide therapeutic benefits while maintaining high engagement and satisfaction levels.

Overall, the cocreation themes guided the iterative refinement of the NoPain Games, confirming they are both engaging and therapeutic for patients with PsA. The integration of feedback from the cocreation session with insights from the relevant literature review resulted in 2 game prototypes that are well-suited to the needs of their target audience, as expressed via the feedback from patients with PsA. In fact, the patient feedback session extended the thematic framework by introducing concrete implementation priorities that reflect lived experience and technical expectations. The mean effective SUS score (79) confirmed good usability, yet the qualitative suggestions revealed areas for refinement that map directly onto the 3 core themes.

First, the recommendation to resolve iOS compatibility issues reinforces theme 3 (accessibility and interaction). While the expert session emphasized touchscreen optimization and inclusive design, patients highlighted platform-specific barriers that could limit access. This aligns with Beltran-Alacreu et al [16], who emphasized the importance of device-level accessibility in digital interventions for chronic pain populations. Addressing cross-platform compatibility is essential to ensure equitable access, particularly for older adults or those with limited digital literacy.

Second, the proposal to incorporate adaptive AI into Space Oddity to tailor difficulty to individual profiles directly advances theme 2 (game difficulty). This feedback moves beyond static balancing and introduces personalization as a therapeutic strategy. It resonates with Tuah et al [20], who identified progression systems and adaptive mechanics as key to sustaining engagement in rehabilitation games. In the context of PsA, where symptom severity and cognitive capacity fluctuate, AI-driven difficulty modulation could enhance both usability and therapeutic relevance.

Third, the suggestion to transition the Four Seasons from prototype to full interactive development with seasonal mechanics and high-quality 3D rendering reflects an evolution of theme 1 (therapeutic benefits). While experts emphasized cognitive stimulation and stress reduction, patients implicitly called for deeper immersion and aesthetic refinement. This aligns with Gromala et al [6], who argued that realistic environments and sensory richness contribute to pain relief. The seasonal metaphor may also support emotional regulation by anchoring gameplay in familiar, cyclical rhythms.

Finally, the idea of integrating smartwatch-based stress tracking introduces a novel extension to Theme 1, bridging subjective experience with physiological feedback. This aligns with recent findings by Pinge et al [46], who systematically reviewed wearable-based stress detection and highlighted the clinical potential of physiological signals such as heart rate variability and electrodermal activity for real-time stress monitoring. By capturing pre- and post-game stress levels, future iterations of NoPain Games could offer biofeedback-informed personalization, enhancing therapeutic precision and enabling longitudinal tracking within digital care ecosystems.

Overall, these patient-driven suggestions not only validate the thematic structure but also push its boundaries toward real-world deployment. They emphasize the importance of technical adaptability, personalized challenge design, and physiological integration in therapeutic game development. More broadly, they demonstrate how participatory feedback can transform conceptual frameworks into actionable design trajectories, ensuring that digital health tools remain grounded in both clinical insight and patient reality.

### Limitations

Although this study offers important insights, it is essential to acknowledge certain limitations. The study engaged a limited sample of 14 experts from 3 European countries in one session, followed by 5 patients with PsA from 4 European countries in a subsequent session, including SUS-based evaluation. While the results offer initial insights into usability perceptions, the limited sample size may have constrained variability, leading to some of the ICC curves being saturated, reducing the ability to detect item-level differentiation. Future research can build on this by involving a broader and more diverse participant pool to deepen understanding of the varied needs and experiences of individuals living with PsA. Particularly, including more testing with patients is necessary, which is essential to the proper design and effectiveness of the games despite the recruiting challenges associated with this task. Disparities in technology literacy among participants, especially health care professionals, also posed a challenge. Providing training or educational materials on the technologies used in the serious games could bridge this gap and improve feedback quality. Moreover, as the study is still at the prototype stage, further testing and clinical validation with patients with PsA through pilot studies and trials will be essential to refine the games based on real-world usability data. Finally, the proposed game designs may not fully consider the physical limitations of patients with PsA, such as joint stiffness and swelling, which can affect usability. Future iterations should incorporate adaptive game design features like customizable controls to improve accessibility.

### Implications

The research on co-designing serious mobile games for patients with PsA presented here has several important implications across various domains.

From a managerial standpoint, the study underlines the importance of interdisciplinary collaboration in developing effective digital health interventions. Managers in health care and technology sectors should foster partnerships between clinicians, researchers, and game developers to leverage their combined expertise. This collaborative approach can lead to more innovative and user-centered solutions [47]. Additionally, the agile co-design methodology highlighted in the study can be adopted by managers to ensure continuous feedback and iterative improvements, enhancing the overall quality and relevance of the developed digital solutions [48]. By promoting a culture of collaboration and iterative development, managers can drive the successful implementation of digital health tools that meet the specific needs of patients [48,49].

From a financial perspective, the development and implementation of NoPain Games for patients with PsA could lead to significant cost savings in health care. By providing an engaging and effective tool for pain management and cognitive stimulation, these games can reduce the reliance on more expensive treatments and interventions [50]. Improved patient adherence to therapeutic activities can also lead to better health outcomes, potentially decreasing the frequency of hospital visits and the need for additional medical support. Furthermore, the scalability of mobile games means that they can be distributed widely at a relatively low cost, making them an economically viable option for health care providers and patients alike [51]. Investing in the development of such digital health solutions can yield long-term financial benefits for both health care systems and patients.

From the clinical point of view, while the NoPain Games presented here are still in the prototype stage and have not yet been used in clinical practice, their potential therapeutic benefits are promising. The proposed serious games are designed to provide pain distraction, cognitive stimulation, and stress reduction, which could significantly improve the quality of life for patients with PsA. Future clinical trials and real-world testing will be crucial to validate these benefits and refine the game mechanics based on patient feedback [52]. If proven effective, these serious games could be integrated into treatment plans as complementary therapy, offering a novel approach to managing chronic pain and enhancing cognitive function. This research also highlights the importance of adaptive difficulty levels and personalized gameplay experiences, which could be further explored in clinical settings to tailor the interventions to individual patient needs [53].

Finally, from a societal perspective, the emphasis on accessibility and inclusive design in the NoPain Games highlights the importance of creating digital health solutions that are usable by individuals with varying physical abilities. By addressing the specific needs of patients with PsA, the games promote greater equity in health care access and support for chronic pain management. This inclusive approach can serve as a model for developing other digital health interventions that address the needs of diverse patient populations [54]. Moreover, the widespread adoption of such games can raise awareness about the potential of serious games in health care, encouraging further innovation and investment in this field [51]. By making effective pain management tools accessible to a broader audience, the research can contribute to improved health outcomes and quality of life for many individuals living with chronic conditions.

In contrast to prior studies that focus on general chronic pain, this research is the first to co-design mobile serious games specifically for patients with PsA, integrating both expert and patient perspectives. It contributes to the field by demonstrating how agile, participatory design can yield tailored, accessible, and therapeutically meaningful digital interventions for a rheumatic population largely overlooked in serious game development.

### Conclusions

The proposed NoPain Games hold significant potential for alleviating chronic pain in individuals with PsA by providing engaging and immersive gameplay experiences aimed at reducing discomfort. This study highlights the collaborative efforts of researchers and clinical/technical experts in designing NoPain Games as cocreators. It introduces 2 serious game prototypes developed using agile methodology and co-design principles, incorporating expert feedback. The study explores the cocreation process, presenting initial findings through storyboards, game visualizations, and prototypes informed by the collected input. Future work includes obtaining further patient feedback on the prototypes and conducting real-world testing to evaluate their feasibility, acceptability, and overall user satisfaction. The findings highlight the critical role of managing game difficulty, which can be addressed through the integration of a dynamic difficulty adjustment system [38,39] in developing the game to customize challenges based on each patient’s condition. Moreover, potential additional features could include integrating smartwatch-based biometric data, such as stress levels and heart rate, to personalize gameplay experiences and provide valuable clinical insights.

## Supplementary material

10.2196/75072 Multimedia Appendix 1 Semistructured guide script for the cocreation session.

10.2196/75072 Multimedia Appendix 2 Online consent form.

## Abbreviations

AI: artificial intelligence
AI-PGS: Artificial Intelligence-Personalized Game Suite
ICC: item characteristic curve
PsA: psoriatic arthritis
SUS: System Usability Scale
UI: user interface
VR: virtual reality
