# Supplementary material for: Co-Designing Mobile Serious Games to Support Patients With Psoriatic Arthritis and Chronic Pain: Mixed Methods Study
Source: JMIR Serious Games. 2026 Jan 30;14:e75072. doi: 10.2196/75072 (PMC12858048; doi:10.2196/75072)
Supplement: Multimedia Appendix 1 [file games-v14-e75072-s001.docx]

## Semi-structured guide script for the co-creation session

**Welcome and Introduction**

- Welcome participants, explain objectives, and request consent to record the session.
- Key question: *“Could you briefly introduce yourself and your role in healthcare?”*

**Recap and Overview**

- Recap key points from the previous co-creation session (i.e., Crazy8s outcomes).
- Present current *NoPain* Games scenarios and emphasize participants’ role.

**Storyboards Presentation**

- Share and explain the proposed *NoPain* Games storyboards, focusing on gameplay mechanics and clinical applications.

**Interactive Panel Discussion.** Key guiding questions:

1. *How do you perceive the potential clinical value of* *the proposed* *NoPain games scenarios?*
2. *Do the presented NoPain game scenarios effectively meet the intended clinical objectives?*
3. *Do you see opportunities to further enhance the user experience, the gameplay, or the clinical effectiveness of the proposed NoPain games?*
4. *Do you have any further comments or recommendations to improve the design of the proposed NoPain game scenarios?*

**Wrap-Up and Next Steps**

- Summarize the collected feedback, outline the next steps for integrating it.

**Closing session**

- Thank participants and encourage them to share additional ideas post-session.
