# Supplementary figures and images for: Co-Designing Mobile Serious Games to Support Patients With Psoriatic Arthritis and Chronic Pain: Mixed Methods Study
Source: JMIR Serious Games. 2026 Jan 30;14:e75072. doi: 10.2196/75072 (PMC12858048; doi:10.2196/75072)

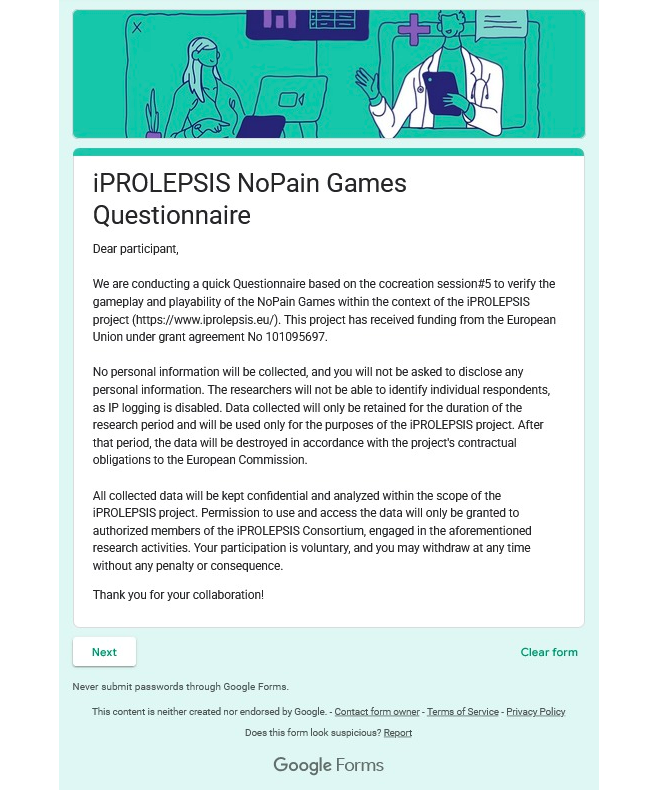

Supplement: Multimedia Appendix 2 [file games-v14-e75072-s002.png]
